# Quality of primary care from patients’ perspective: a cross sectional study of outpatients’ experience in public health facilities in rural Malawi

**DOI:** 10.1186/s12913-018-3701-x

**Published:** 2018-11-20

**Authors:** Luckson Dullie, Eivind Meland, Thomas Mildestvedt, Øystein Hetlevik, Sturla Gjesdal

**Affiliations:** 1 Department of Global Public Health and Primary Care, University of Bergen, Bergen, Norway; 2 Partners In Health, Blantyre, Neno Malawi; 3 University of Malawi College of Medicine, Blantyre, Malawi

**Keywords:** Primary care, Primary care performance, Primary care assessment tool, Patient experience measurement, Health services, Malawi

## Abstract

**Background:**

Assessing patients’ experience with primary care complements measures of clinical health outcomes in evaluating service performance. Measuring patients’ experience and satisfaction are among Malawi’s health sector strategic goals. The purpose of this study was to investigate patients’ experience with primary care and to identify associated patients’ sociodemographic, healthcare and health characteristics.

**Methods:**

This was a cross sectional survey using questionnaires administered in public primary care facilities in Neno district, Malawi. Data on patients’ primary care experience and their sociodemographic, healthcare and health characteristics were collected through face to face interviews using a validated Malawian version of the primary care assessment tool (PCAT-Mw). Mean scores were derived for the following dimensions: first contact access, continuity of care, comprehensiveness, community orientation and total primary care. Linear regression models were used to assess association between primary care dimension scores and patients’ characteristics.

**Results:**

From 631 completed questionnaires, first contact access, relational continuity and comprehensiveness of services available scored below the defined minimum. Sex, geographical location, self-rated health status, duration of contact with facility and facility affiliation were associated with patients’ experience with primary care. These factors explained 10.9% of the variance in total primary care scores; 25.2% in comprehensiveness of services available and 29.4% in first contact access.

**Conclusion:**

This paper presents results from the first use of the validated PCAT-Mw. The study provides a baseline indicating areas that need improvement. The results can also be used alongside clinical outcome studies to provide comprehensive evaluation of primary care performance in Malawi.

## Background

Measuring patients’ experience with care should be part of the process of establishing services and delivering primary care that users need [1]. This facilitates understanding of gaps [2], informs health authorities on trends of quality of care [3], and ensures transparency and accountability [4]. Patient experience is also an important measure of healthcare quality [5, 6] and positive experiences are associated with better health outcomes [7].

Malawi’s health sector strategic plan for 2017 to 2022 is based on principles of primary health care and aspires for patient satisfaction [8]. The country has recently registered notable progress especially in HIV/AIDS and child health indicators [9]. However significant challenges still remain including severe shortage of healthcare workers [10], access, equity [11] and protection of vulnerable people from catastrophic financial burden borne in the course of seeking healthcare even though public services are free at the point of care [12].

Malawi does not have a specific primary care policy that defines the gate-keeping role of primary care. However, patients enter the public health system through a primary care level staffed by nurses and mid-level provider medical assistants. Primary care facilities refer patients to district hospitals where, in addition to the mid-level providers, there are two to three physicians typically without any specialization. Tertiary hospitals are located in four regions of the country.

Neno, is a rural district with an estimated population of 170,000. The district is supported by the international non-governmental organization Partners In Health (PIH) to develop a model of district health services. There are two hospitals and seven health centers under the Ministry of Health; four health centers under a faith-based organization and one health center largely for employees of an electricity generation company. Faith-based health facilities charge user fees. With support from PIH, Neno has the highest per capita health funding in Malawi at nearly 66 US$ [13] compared to the national average of 30 US$ [8]. The additional resources are used to hire extra healthcare workers and procure supplementary medical supplies. Recent studies from Neno show better health outcomes in maternal and child mortality [13], HIV care [14, 15], Kaposi sarcoma and palliative care [16], and financial risk protection for vulnerable patients [17]. In addition, innovative primary care approaches have been implemented in non-communicable diseases [18, 19]and an extensive structure of community health workers is supporting the health system [20].

## Methods

The aim of this study was to evaluate the performance of primary care in Neno based on patients’ experience of services. Specifically, the study measured the performance of primary care in Neno through total primary care and dimension mean scores and assessed association between the scores and patients’ sociodemographic, healthcare and health characteristics.

### The instrument

Within primary health care research, the US Primary Care Assessment Tool (PCAT) has been widely adapted and used in patient surveys in many countries [21–26]. Based on the 1994 American Institute of Medicine’s definition of primary care [27] the PCAT aims at a global assessment of primary care organization and its performance in the core dimensions of accessibility, comprehensiveness, coordination and continuity, and accountability. In addition, it also assesses derivative dimensions of family orientation, community orientation, and cultural competence.

The development and validation of the Malawian version of the primary care assessment tool (PCAT-Mw) has been documented in another paper [28]. The tool has 29 items (Table 1) measuring primary care performance in seven dimensions: first contact access (3 items), communication continuity of care (4 items), relational continuity of care (4 items), coordination (3 items), comprehensiveness of services available (6 items), comprehensiveness of services provided (6 items) and community orientation (3 items). First contact access is here defined as the manner in which services are organized to accommodate access whenever needed and ensure patient satisfaction. Continuity of care entails the existence of a regular source of care and the longitudinal relationship between primary care providers and patients, in terms of accommodation of patient’s needs and preferences, such as communication and respect for patients. Coordination of care reflects the ability of primary care providers to facilitate and support patients to navigate use of other levels of health care when needed. Comprehensiveness of primary care services represents the range of services available in primary care to meet patients’ health care needs. A distinction is made between services that are available and those that are actually provided. Community orientation defines the extent to which the primary care providers understand and address priority health problems in a particular community with evidence of community participation.

**Table 1:** Validated questionnaire items of the PCAT-Mw

First contact access (3 items)	
1. When this HC is closed on Saturday and Sunday and you get sick, would someone from here see you the same day?	
2. When the HC is closed and you get sick during the night, would someone from here see you that night?	
3. Is there a complaints / suggestion box at this HC?	
Communication continuity of care (4 items)	
1. Is the staff friendly and approachable?	
2. Do you think the staff at this HC understands what you say or ask?	
3. Are your questions answered in a way that you understand?	
4. Does this HC give you enough time to talk about your problems or worries?	
Relational continuity of care (4 items)	
1. Does this HC know you very well as a person, rather than as someone with a medical problem?	
2. Does this HC know who lives with you?	
3. Does this HC know your complete medical history?	
4. Does this HC know about your work or employment?	
Coordination (3 items)	
1. Does this HC know what the results of the visit were?	
2. After you went to the specialist or hospital, did this HC talk with you about what happened at that visit?	
3. Does this HC seem interested in the quality of care that you get from that specialist or hospital?	
Comprehensiveness of services available (6 items)	
1. Checking hearing	
2. Dental check-up – checking and cleaning your teeth	
3. Treatment by dental therapist eg extraction of bad teeth	
4. Counseling for mental health problems	
5. Plastering of fractures	
6. Treatment of ingrown toe nails or removing part of a nail	
Comprehensiveness of services provided (6 items)	
1. Advice on wearing reflectors when walking on the road at night	
2. How to prevent hot burns	
3. Advice about appropriate exercise for you	
4. Advice on how to prevent accidental falls	
5. Ways to handle family conflict; arguments; disagreements (that may arise from time to time)	
6. Possible exposure to harmful substances in your home, at work or in your area e.g. paraffin; pesticides?	
Community orientation (3 items)	
1. Do you think this HC knows about the important health problems of your area?	
2. Does this HC get opinions and ideas from people or organizations with knowledge to help provide better health care? E.g. the local health committee, churches, other organizations?	
3. Does this HC do surveys of patients to see if services are meeting the needs of the people?	

Items are scored on a 4-point Likert scale, with 1 indicating “definitely not,” 2 indicating “probably not,” 3 representing “probably,” and 4 representing “definitely.” For consistency with methods used in PCAT studies in other countries, a mid-scale value of 2.5 is assigned to “not sure” answers while the mean item score is used for missing data [22–24]. Additionally, there are questions to identify the usual primary care facility the patient uses and the patient’s sociodemographic data. This paper excludes the 3 coordination items because insufficient number of patients had been referred for secondary level care.

### Setting, study population and data collection

A face to face administered cross sectional study was carried out in August –September, 2016 in outpatient clinics of ten facilities – the two hospitals and eight health centers in Neno district. Facilities were selected purposefully to include all the public health facilities in the district. One of the faith-based health centers was included as it had signed a memorandum of understanding with the authorities to remove the user fees and run as a public facility. Patients were at least 18 years of age, must have used the facility for at least six months and must have visited the facility for at least 3 times. Acutely ill, frail looking or severe mental health patients were excluded in order to allow them to receive urgent medical attention. As this study’s data collection was part of the validation of the PCAT-Mw through metric analyses, sample size was calculated based on similar studies using at least 5:1 subject to item ratio [22–26]. Sample size of 600 was targeted, 60 from each facility.

Six interviewers were trained to conduct the PCAT-Mw survey. A pilot study showed that the questionnaire would take about 45 min to administer. Each interviewer was therefore expected to interview seven patients per day. The sampling frame was 50–60 patients waiting to be seen on each working day. Sampling interval (n) was calculated by dividing the number of waiting patients by seven. A random starting point was obtained using a smart phone random number generator. Each ‘n^th^’ patient was then asked for consent to participate in the study.

### Sociodemographic, health care and health measures

Independent variables were sex, age, education, geographical location, duration of contact with facility, reason for attending: chronic or acute condition, distance to facility measured through time taken to walk to the facility, cost of travel to the facility, waiting time, individual health facility affiliation and self- rated health status.

### Statistical analysis

Data were entered into and analyzed using the IBM SPSS Statistics 24.0.0 (2016) package. Dimension mean scores were derived by dividing the sum of the item means by the number of items in the dimension. A score ≥ 3 was considered ‘acceptable to good performance’ and < 3 as ‘poor performance’. [28, 29] Total primary care was calculated as the sum of all dimension means. Sociodemographic, health care and health characteristics of the patients were compared between sexes by performing cross table analyses with chi squared significance testing to highlight differences between male and female patients.

Next, independent sample T tests were done to compare dimension means and total primary care scores between the sexes. Multiple linear regression models were used to assess association between sociodemographic, health care and health characteristics and total primary care scores after adjusting for sex and age. Further, stepwise exclusion regression models were used to identify independent variables that accounted for significant variances in patients’ experiences with regard to total primary care and individual dimension mean scores. For all tests, confidence intervals of 95% and a *p*-value less than 0.05 were used as thresholds of statistical significance.

## Results

### Patients’ characteristics

A total of 649 patients were approached and 18 (2.8%) declined to participate in the study. This paper presents results from 631 completed questionnaires. Missing data accounted for approximately 1.9% of all data. Table 2 compares sociodemographic, health care and health characteristics of study participants between sexes. Overall, 65.0% of primary care visits were from female patients. (Table 2: Sociodemographic, health care and health characteristics among 631 patients attending outpatient clinics in Neno district, Malawi in August and September, 2016 compared between sexes).

**Table 2:** Sociodemographic, health care and health characteristics among 631 patients attending outpatient clinics in Neno district, Malawi in August and September, 2016 compared between sexes

Characteristic	Female (*n* = 410) (%)	Male (*n* = 221) (%)
Age		
18–30 years	197 (48.0)	73 (33.0)
31–45 years	152 (37.1)	94 (42.6)
Above 45	61 (14.9)	54 (24.4)**
Education		
None	48 (11.7)	12 (5.5)
Up to 5 years primary	153 (37.3)	58 (26.2)
5–8 years primary	145 (35.4)	95 (43.0)
At least secondary	64 (15.6)	56 (25.3)**
Duration of contact with facility		
Up to 2 years	66 (16.1)	27 (12.2)
2–4 years	88 (21.5)	41 (18.6)
> 4 years	256 (62.4)	153 (69.2)
Time to walk to facility		
< 1 h	198 (48.3)	136 (61.5)
≥ 1 h	212 (51.7)	85 (38.5)*
Cost of travel to facility#		
0 MK	299 (73.9)	143 (64.7)
Up to 500 MK	45 (11.0)	17 (7.7)
> 500 MK	66 (.15.1)	61 (27.6)*
Waiting time at facility		
Up to 30 mins	167 (40.7)	69 (31.2)
30–90 min	136 (33.2)	81 (36.7)
> 90mins	107 (26.1)	71 (32.1)
Reason for attending facility		
Chronic condition	161 (39.3)	89 (40.3)
Acute condition	249 (60.7)	132(59.7)
Self-rated health status		
Poor to fair	129 (31.5)	83 (37.6)
Good	60 (14.6)	36 (16.3)
Very good to excellent	221 (54.0)	102(46.1)
Geographical location		
Upper Neno	153 (37.3)	106 (48.0)
Lower Neno	257 (62.7)	115 (52.0)*

Chi squared *p* value * < 0.01

**< 0.001

# 500MK is close to US$0.75

### Primary care dimension scores

Table 3 shows poor performance in relational continuity (2.3), comprehensiveness of services available (2.4) and first contact access (2.8). The highest score was in communication continuity of care (3.6). Community orientation and comprehensiveness of services provided also achieved acceptable performance at 3.1 and 3.2 respectively. Female patients scored lower than male patients in all dimensions but the difference was significant only in total primary care (*p* = 0.01), first contact access (*p* = 0.021), relational continuity (*p* = 0.044) and comprehensiveness of services available (*p* = 0.017).

**Table 3:** Primary care dimension mean scores among patients attending outpatient clinics in Neno district in August–September, 2016 compared between the total sample (*N* = 631), male (n = 221) and female patients (*n* = 440)

Primary care dimension	Number of items	Mean scores (SEM)		
		Total	F	M
Sample size		631	410	221
First contact access	3	2.8 (0.03)	2.8 (0.04)	2.9 (0.05)*
Communication continuity	4	3.6 (0.02)	3.6 (0.03)	3.6 (0.04)
Relational continuity	4	2.3 (0.04)	2.2 (0.05)	2.4 (0.07)*
Comprehensiveness				
Services available	6	2.4 (0.03)	2.4 (0.04)	2.5(0.06)*
Services provided	6	3.2 (0.04)	3.1 (0.04)	3.2(0.06)
Community orientation	3	3.1 (0.04)	3.1 (0.05)	3.1(0.07)
Total primary care score	26	17.4 (0.12)	17.2 (0.15)	17.7 (0.21)*

Independent sample T-test *p* values: * < 0.05

### Multivariate analyses

(Table 4: Linear regression models assessing association between sociodemographic and health care factors and total primary care scores with unstandardized beta values among 631 patients attending outpatient clinics in Neno district, Malawi (August–September, 2016). Table 4 presents the linear regression models assessing association between patient characteristics and total primary care scores. Male patients scored 0.7 points higher than females (95% CI = 0.2, 1.2; p = 0.01). After adjusting for sex and age, patients in upper Neno scored total primary care 0.5 points higher than lower Neno patients (95% CI = 0.04, 1.0; *p* = 0.033). Increasing self-rated health status (rated on a 5 point Likert scale from very poor to excellent) was associated 0.8 points higher scores at good (95% CI = 0.1, 1.5; *p* = 0.034) and 0.9 points for very good to excellent (95% CI = 0.3, 1.4; *p* = 0.002), duration of contact with facility of more than 4 years was associated with scores 1.1 points higher (95%CI = 0.4, 1.2; *p* = 0.003) while acute presentation was associated with 0.6 points lower (95% CI = − 1.0, − 0.1; p = 0.03). At the individual facility level, patients from the health centers scored significantly below the reference outpatient clinic at the district hospital by points ranging from 0.6 to 2.0. Level of education, distance to the facility, cost of travel to the facility and waiting time were not associated with total primary care scores.

**Table 4:** Linear regression models assessing association between sociodemographic and health care factors and total primary care scores with unstandardized beta values among 631 patients attending outpatient clinics in Neno district, Malawi (August–September, 2016)

Factor	B	95%CI	*P* value
Sex^a^			
Female^c^	17.1	16.8, 17.4	
Male	0.7	0.2, 1.2	0.01
Age^a^			
18–30 years^c^	17.2	16.8, 17.6	
30–45 years	0.2	− 0.3, 0.8	0.43
> 45 years	0.4	− 0.3, 1.1	0.24
Education^b^			
0–5 years primary^c^	17.0	16.5, 17.4	
6–8 years primary	0.3	− 0.2, 0.9	0.23
At least secondary	−0.4	−1.1, 0.3	0.28
Geographical location^b^			
Lower Neno^c^	16.8	16.4, 17.3	
Upper Neno	0.5	0.04, 1.0	0.033
Distance to facility^b^			
< 1 h walk^c^	16.9	16.5, 17.4	
> 1 h walk	0.2	−0.3, 0.7	0.38
Cost of travel to facility^b^			
0 MK^c^	17.1	16.7, 17.5	
1–500 MK	− 1.0	−1.8, − 0.2	0.016
> 500 MK	0.2	−0.4, 0.8	0.57
Waiting times at facility			
Up to 30 mins^c^	17.0	16.5, 17.5	
30–90 min	− 0.3	−0.9, 0.3	0.31
> 90 mins	0.4	−0.2, 1.0	0.20
Duration of contact^b^			
Up to 2 years^c^	16.3	15.7, 17.0	
2–4 years	0.3	−0.5, 1.2	0.42
> 4 years	1.1	0.4, 1.2	0.003
Reason for attendance^b^			
Chronic condition^c^	17.4	16.9, 17.9	
Acute condition	−.0.6	−1.0, − 0.1	0.03
Self-rated health status^b^			
Poor – fair^c^	16.4	15.8, 16.9	
Good	0.8	0.1, 1.5	0.034
> good	0.9	0.3, 1.4	0.002
By health facility^b^			
A^c^ (hospital outpatient clinic)	18.3	17.5, 19.1	
B (health center)	−1.2	−1.2, −0.2	0.018
C (health center)	−0.6	−1.6, 0.5	0.30
D (health center)	−1.5	−2.5, −0.4	0.006
E (health center)	−1.6	−2.7, −0.6	0.002
F (hospital outpatient clinic)	0.5	−0.53, 1.51	0.34
G (health center)	−2.0	−3.1, −1.0	< 0.001
H (health center)	−1.7	−2.8, −0.7	0.001
I (health center)	−2.0	−3.0, −1.0	< 0.001
J (health center)	−1.5	−2.7, −0.4	0.01

^a^unadjusted linear regression models

^b^linear regression models adjusted for sex and age

^c^Reference

(Table 5: Association between predictors and total primary care scores, access and comprehensiveness of services available mean scores with unstandardized beta values among 631 patients attending outpatient clinics in Neno, Malawi (August – September, 2016)). The investigated factors explained 10.9% of the noted variance in total primary care scores. Looking at each dimension, these sociodemographic and health care characteristics explained 29.4% of variance in first contact access and 25.2% in comprehensiveness of services available (Table 5). These factors also explained 3% of variance in comprehensiveness of services provided, 3.7% in community orientation, 4.4% in relational continuity of care and 5.2% in communication continuity of care (data not shown in the table).

**Table 5:** Association between predictors and total primary care scores, access and comprehensiveness of services available mean scores with unstandardized beta values among 631 patients attending outpatient clinics in Neno, Malawi (August – September, 2016)^a^

	B	95% CI	*p* value
Model 1: Total primary care scores			
Reference	15.8	15.1, 16.4	
Facility F	2.3	1.6, 3.1	< 0.001
Upper Neno	0.9	0.4, 1.4	< 0.001
Self-rated health = good	1.1	0.3, 1.3	< 0.001
Duration of contact > 4 years	0.8	0.6, 1.7	0.001
Education >at least secondary	−0.8	−1.3, −0.2	0.011
Self -rated health = very good/excellent	0.9	0.2, 1.6	0.013
Acute presentation	−0.6	−1.1, − 0.1	0.017
Male sex	0.5	0.03, 1.0	0.036
Unadjusted R^2^	12.1%		
Adjusted R^2^	10.9%		
Model 2 First contact access dimension scores			
Reference	2.9	2.9, 3.1	
Facility F	0.8	0.8, 1.0	< 0.001
Facility G	−0.8	−0.8, −0.6	< 0.001
Facility H	−0.6	−0.6, − 0.4	< 0.001
Facility I	− 0.3	− 0.3, − 0.1	0.001
chronic condition	−0.2	− 0.2, − 0.1	0.003
Cost of travel >MK500	0.1	0.1, 0.3	0.047
Unadjusted R^2^	30.1%		
Adjusted R^2^	29.4%		
Model 3 Comprehensiveness of services available dimension sum scores			
Reference	2.0	1.9, 2.2	
Upper Neno	0.9	0.7, 1.1	< 0.001
Facility B	1.2	1.0, 1.5	< 0.001
Facility C	−1.2	−1.5, −1.0	< 0.001
Facility D	−1.1	− 1.4, −0.9	< 0.001
Facility F	−0.9	− 1.1, − 0.7	< 0.001
Education >at least secondary	− 0.2	− 0.4, − 0.1	0.002
Travel time > 1 h	0.2	0.03, 0.3	0.012
Self -health rating = very good/excellent	0.1	0.01, 0.2	0.04
Unadjusted R^2^	26.1%		
Adjusted R^2^	25.2%		

^a^Multivariate regression with stepwise exclusion method where significant predictors are retained in the models

## Discussion

To our knowledge, this paper is the first time primary care performance has been measured based on patients’ experience in Malawi. The study shows poor performance in relational continuity, comprehensiveness of services available and first contact access. Acceptable performance was achieved in community orientation, comprehensiveness of services provided, and communication continuity of care.

The study shows that more primary care visits were from female patients; who also tended to have lower levels of education similar to findings in a South African study [29]. The female patients in this study also rated their primary care experience lower than male patients. Literature review of health-seeking behavior studies shows that women consult more frequently than men [30]. Since the women in this study were younger, reproductive health reasons might at least partially explain the gender difference as was the case in a UK study [31]. Further studies are needed to understand this difference in primary care experience in the Malawi context in order to better inform options for interventions to close the gap such as more comprehensive sexual and reproductive services.

Most public primary care facilities in Malawi serve a geographically recognizable catchment population. This provides opportunity for relational continuity of care and population based primary care approaches. Population management, stable patient-team partnership, and continuity of care are known building blocks of effective primary care systems [32] This study shows that most patients had affiliation with their public primary care facilities for at least 4 years. Duration of contact of four years or longer was associated with higher total primary care scores but the direction of the association cannot be ascertained in this study. Relational continuity was poor and as such was one of the areas that need further exploration and improvement.

Most patients’ reason for their primary care visit in this study was care for acute conditions. However, care for chronic conditions was associated with better overall experience. Chronic care patients were given appointments for their visits and were usually attended by the same team. Community health workers also followed up patients when they missed their appointments. Further prospective studies should be carried out to assess if these processes of care would explain the differences and if the primary care experience of patients presenting with acute conditions would improve when offered the same management.

Health centers play an important gate-keeping role that is essential to well-functioning health systems. This is not clearly defined in Malawi’s district health system although patients are expected to first report to their public primary care facilities by virtue of proximity. In this study, health centers were scored lower than the outpatient clinics at the hospitals with regard to total primary care, first contact access and comprehensiveness of services available. A study in several African countries showed that staffing levels, experience of providers and facility management were associated with quality of care provided [33]. While there is need to investigate factors that would account for this variation at facility level, the gate-keeping function of health centers could be enhanced both through clear policy formulation as well as interventions such as providing better qualified staff, and paying more attention to facility management to improve access to quality and comprehensive package of services in the public health centers.

Users who rated their health status as ‘good’ or ‘very good’ also rated primary care experience better than those who rated their health as ‘poor’. Similar findings have been reported in the Korean and South African PCAT studies [24, 29]. Although it is possible that those who reported better health had actually benefited from the care itself, the direction of the association cannot be ascertained through a cross sectional study such as this.

Education, age, distance to facility and cost of travel were not associated with total primary care scores. A lack of association between socioeconomic factors and patients’ experience of primary care has also been reported in other studies. [24, 29, 34] This might be ascribed to the robustness of the questionnaire to accurately measure users’ primary care experience independent of differences among patients such as age, gender, poverty or educational levels.

Low scores noted in first contact access, comprehensiveness of services available and relational continuity of care are similar to findings in other studies [29, 34]. In Malawi, this is likely related to acute shortage of staff especially in primary care, inadequate staff training and lack of equipment and supplies particularly at health centers.

The factors that were significantly associated with patients’ experience of primary care accounted for much higher variances in first contact access and comprehensiveness of services provided dimensions, 29.4 and 25.2% respectively. This underscores the importance of access and availability of services as the core factors on which the other dimensions of primary care depend. Utilization, continuity, coordination and service provision will take place successfully only when people have effective access to facilities and services that they need which is an important objective of universal health coverage [35]. Improved primary care will therefore require multi-level interventions to address these gaps and countries need to translate political will into action in order to attain primary care for all.

### Strengths and limitations

Strengths of the study include use of a globally accepted tool that had been culturally adapted and validated for use in Malawi [28]. The PCAT-Mw has advantages compared to other tools that measure patient perspectives in that it assesses patient experience with care. Since this is the first time the PCAT-Mw has been applied in a clinical setting in Malawi, the results of the paper provide a measure of the performance of primary care in Malawi. This also adds to the construct validation of the questionnaire.

The study had a number of limitations. First, because this was a cross-sectional study, causal inferences to findings are not possible. Second, liability to several types of bias is noted: recall, response and selection. The face to face interview partly minimized recall bias through clarifying questions whenever that was necessary. Potential for response bias was possible because data collection was done during clinic visit. Selection bias might have resulted from excluding those who were acutely ill, frail or had severe mental illness and interviewing only patients who attended clinics and might have had better experience than the patients excluded. The study was also carried out in one district only. In another subsequent study, we have included multiple sites to improve generalizability of results. Third, the factors identified accounted for 10.9% of total primary care score variances, 25.2% in the comprehensiveness of services available and 29.4% in the first contact access. Potential unmeasured factors such as the actual quality of services provided and health care workers’ skills, attitude and behaviors might confound the results. Fourth, this was a study of patient experiences of primary care and not of health outcomes. Further studies could assess correlations between clinical outcomes and patient experiences of care and the extent to which patient experiences predict later health outcomes.

## Conclusions

This paper presents results from the first use of the validated PCAT-Mw to assess patients’ experience of primary care and associated sociodemographic, health care and health factors in a rural district in Malawi. Patients reported acceptable levels of performance in the primary care dimensions of communication continuity of care, comprehensiveness of services provided and community orientation. Poor performance was reported in first contact access, comprehensiveness of services available and relational continuity of care. Our experience indicates that the PCAT-Mw can be used alongside clinical health outcome studies to provide.

comprehensive evaluation of primary care performance in Malawi. The areas of poor patient experience need further research to evaluate possible explanations and to inform appropriate interventions.

## Abbreviations

PCAT - Mw: Primary Care Assessment Tool – Malawian version
PCAT: Primacy Care Assessment Tool
PIH: Partners In Health
